# Targeted and genome-wide sequencing reveal single nucleotide variations impacting specificity of Cas9 in human stem cells

**DOI:** 10.1038/ncomms6507

**Published:** 2014-11-26

**Authors:** Luhan Yang, Dennis Grishin, Gang Wang, John Aach, Cheng-Zhong Zhang, Raj Chari, Jason Homsy, Xuyu Cai, Yue Zhao, Jian-Bing Fan, Christine Seidman, Jonathan Seidman, William Pu, George Church

**Affiliations:** 1 Department of Genetics, Harvard Medical School, Boston, Massachusetts 02115, USA; 2 Wyss Institute for Biologically Inspired Engineering, Harvard University, Boston, Massachusetts 02115, USA; 3 Department of Cardiology, Boston Children’s Hospital, Boston, Massachusetts 02115, USA; 4 Broad Institute of MIT and Harvard, Cambridge, Massachusetts 02142, USA; 5 Illumina, San Diego, California 92122, USA

## Abstract

CRISPR/Cas9 has demonstrated a high-efficiency in site-specific gene targeting. However, potential off-target effects of the Cas9 nuclease represent a major safety concern for any therapeutic application. Here, we knock out the *Tafazzin* gene by CRISPR/Cas9 in human-induced pluripotent stem cells with 54% efficiency. We combine whole-genome sequencing and deep-targeted sequencing to characterise the off-target effects of Cas9 editing. Whole-genome sequencing of Cas9-modified hiPSC clones detects neither gross genomic alterations nor elevated mutation rates. Deep sequencing of *in silico* predicted off-target sites in a population of Cas9-treated cells further confirms high specificity of Cas9. However, we identify a single high-efficiency off-target site that is generated by a common germline single-nucleotide variant (SNV) in our experiment. Based on *in silico* analysis, we estimate a likelihood of SNVs creating off-target sites in a human genome to be ~1.5–8.5%, depending on the genome and site-selection method, but also note that mutations might be generated at these sites only at low rates and may not have functional consequences. Our study demonstrates the feasibility of highly specific clonal *ex vivo* gene editing using CRISPR/Cas9 and highlights the value of whole-genome sequencing before personalised CRISPR design.

## Abstract (web summary)

The microbial RNA-guided CRISPR/Cas9 system has robust genome-editing activities, but the off-target effects of the Cas9 nuclease have only recently begun to be analysed. Here the authors provide evidence for high specificity of the Cas9 nuclease on targeting of the Tafazzin gene in human-induced pluripotent stem cells and demonstrate the impact of single-nucleotide variations of the human genome on Cas9 specificity.

## Graphical abstract

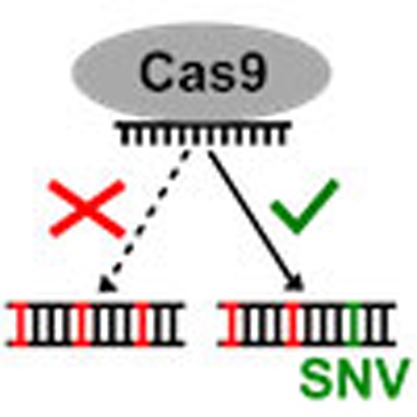

The recent engineering of CRISPR/Cas9 (ref. 1), a bacterial-adaptive immune system, has reshaped the field of molecular biology. The complex of a customised guide RNA (gRNA) and the nuclease Cas9 enables specific recognition and cutting in the gRNA-target region upstream of a protospacer adjacent motif (PAM; NGG for *S. pyogenes* Cas9). As a programmable genome-engineering tool^2^,^3^, CRISPR/Cas9 system offers several advantages over previous sequence-specific nucleases and has been demonstrated to have robust genome-editing activities in over 20 organisms^4^,^5^,^6^,^7^.

Although CRISPR/Cas9 has demonstrated a high-efficiency of target-site modification, for clinical applications such as gene therapy, off-target Cas9-nuclease activity is a major concern. Off-target genomic alterations can have fatal outcomes such as disrupting essential genes or generating chromosomal rearrangements^8^. Even though deep sequencing of *in silico* predicted off-target sites in the genome^9^,^10^ and expression-based reporter assays^11^ have shown Cas9 to be specific, these approaches overlook the possibility of unpredictable off-targets and potential disruption of genome integrity. In particular, subtle differences due to genetic variants are not considered in reference genome based off-target site prediction.

Here we examine Cas9 specificity through a combination of whole-genome sequencing of clones derived from single Cas9-modified human-induced pluripotent stem cells (hiPSCs) and deep sequencing of predicted off-target sites in a population of hiPSCs. We demonstrate that Cas9-modified hiPSC clones do not exhibit elevated mutation rates and that Cas9 nuclease has high mismatch sensitivity, which is consistent with recent publications^12^,^13^. The low frequency of off-target Cas9 activity suggests that it is feasible to screen for single-cell derived hiPSC clones with specific gene targeted and minimal off-target effects for clonal *ex vivo* applications. In addition and in contrast to the previous studies, we first observe that a common single-nucleotide variant (SNV) in the human genome can create a high-efficiency Cas9 off-target site. We estimate the practical range of probabilities of SNVs creating high probable off-target sites to be ~1.5–8.5%, depending on the genome and method of site selection. We however also note that for clinical purposes site selection methods will be used that yield lower probabilities of SNV-generated off-targets. Based on our results, we conclude that personal whole-genome characterisation is advisable to achieve specific gene editing using Cas9.

## Results

### Cas9 activity induces deletions at the target site

We chose to target the *Tafazzin* (*TAZ*) gene on the X chromosome. *TAZ* mutations have been associated with a number of cardiovascular disorders such as Barth Syndrome^14^.

**Figure 1:**
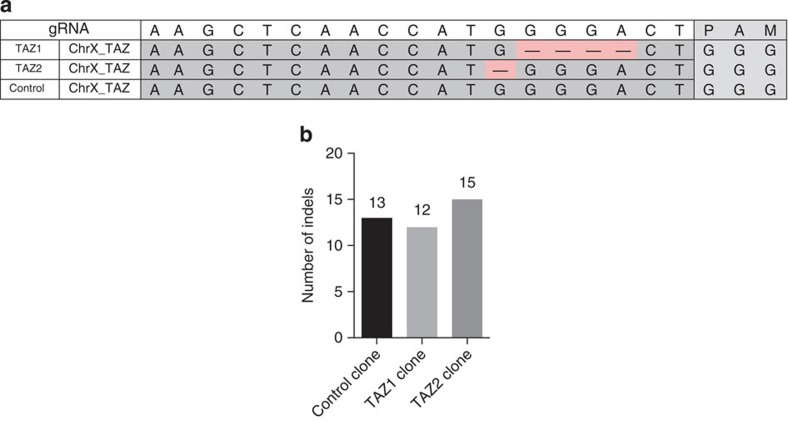
Cas9 activity does not increase the rate of indels above background. (**a**) Frameshift deletions are introduced in the TAZ gene of two single-cell derived colonies TAZ1 and TAZ2; (**b**) Number of *de novo* indels in the control clone and TAZ1 and TAZ2 clones as detected by whole-genome sequencing analysis.

To target the *TAZ* gene, we integrated Cas9 under control of Tet-On transactivator into the genome of PGP1 (ref. 15) hiPSCs via the *PiggyBac* transposon and transfected the cells with a gRNA targeting a region (chr.X: 153,647,923–153,647,944) close to the mutation found in Barth Syndrome patients^16^. Genomic integration of Cas9 was chosen over transient transfection to ensure maximal Cas9 activity and enable high detection sensitivity. The control sample was transfected with a gRNA that has no similar site (≥15 bp of 20 bp) in hg19 reference genome (Supplementary Fig. 1). Single-cell derived hiPSC colonies were genotyped for on-target activity and the pluripotent state was verified through quantification of *OCT4* and *NANOG* expression levels (Supplementary Figs 2 and 3). Two *TAZ*-knockout clones (TAZ1 and TAZ2) and one control clone were whole-genome sequenced. Short deletions at the target site were observed in both the *TAZ*-knockout clones as a result of Cas9 activity (Fig. 1a). No modification at the *TAZ* target site was observed in the control clone (Fig. 1a).

### Cas9 activity does not increase the mutation rate

To comprehensively investigate the off-target effects of Cas9, we searched for *de nov*o mutations in the whole-genome sequencing data.

As Cas9-induced DNA double-strand breaks are primarily repaired *via* non-homologous end-joining to yield small insertions or deletions, we first performed a genome-wide search of sample-specific insertion/deletions (indels) through comparative analysis of *TAZ*-knockout clones versus the control clone and excluded shared-germline variants or mutations that were acquired before Cas9 treatment. Both *TAZ*-knockout samples as well as the control sample showed a comparable number of *de novo* indels in the genome (Fig. 1b), suggesting minimal mutagenesis due to Cas9 activity. We further performed exome sequencing of two additional *TAZ*-knockout clones and identified no indels in one clone (TAZ3) and one indel in the second clone (TAZ4) (Supplementary Fig. 4). From these results we conclude that targeted Cas9 activity does not increase the genome-wide indel rate.

We further asked if Cas9-induced DNA double-strand breaks could lead to large-scale genomic alterations^17^. To this end, we examined the number of *de novo* genomic rearrangements and found very few rearrangements and no large-scale copy-number alterations (>100 kb) in either clone (Table 1, Supplementary Fig. 5). The genomic integrity was also validated through karyotyping (Supplementary Fig. 6).

Together, these results demonstrate that Cas9 does not elevate mutation rates on the genome-wide scale or compromise genome integrity.

### A germline SNV creates a single recurrent off-target site

**Figure 2:**
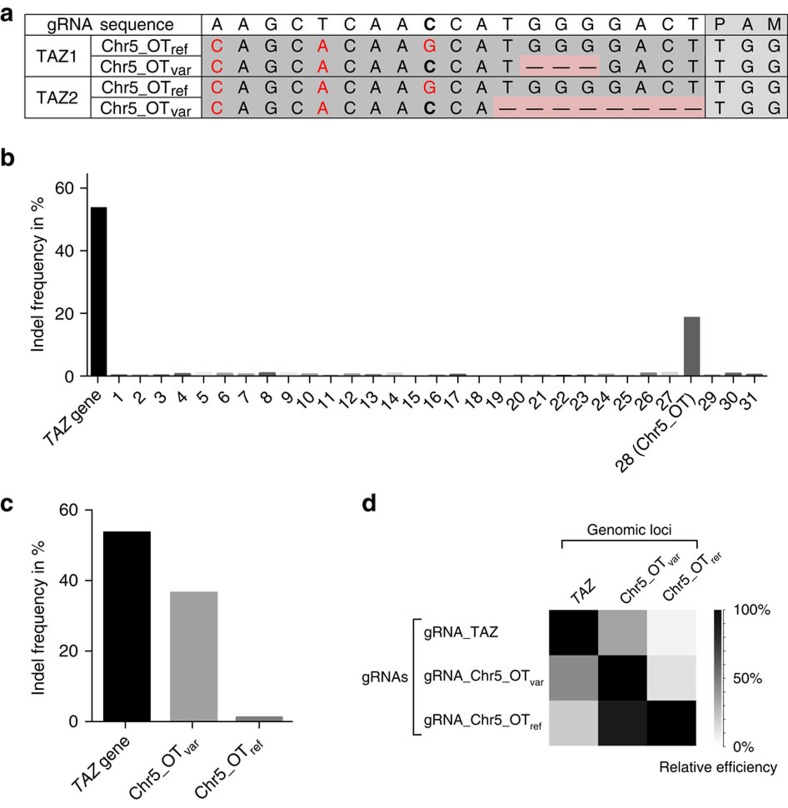
A germline SNV creates a single high-efficiency off-target site. (**a**) A heterozygous-common SNV at the Chr5_OT site in the genome of PGP1 cells creates a genomic allele with only two mismatches to the TAZ-targeting gRNA (Chr5_OT_var_). Chr5_OT_var_ was targeted in both TAZ-clones, as evidenced by the small-deletions detected by WGS, while the reference allele Chr5_OT_ref_ with three mismatches remained intact. (**b**) Deep sequencing of 31 (out of 32) potential off-target sites with at the most three mismatches revealed Chr5_OT as the only high-efficiency off-target site. Sequences of the predicted off-target sites and the indel frequencies for each site can be found in Supplementary Tables 1 and 2. (**c**) Deep sequencing revealed the allele-specific nature of Cas9. Cas9 demonstrated high-targeting efficiencies at the TAZ-target site and the Chr5_OT_var_ allele but minimal efficiency at Chr5_OT_ref_ as measured by the indel frequency. The indel frequency at Chr5_OT_ref_ was in the same range as indel frequencies at all other three-mismatch off-target sites (Supplementary Table 2). (**d**) Varying the gRNA sequence relative to the target-site sequence confirmed the high-mismatch sensitivity of Cas9. The pairwise targeting efficiency between each gRNA and each target site was measured by the indel frequencies detected by deep sequencing. Relative efficiencies were calculated by normalising the off-target indel frequencies to the on-target frequencies.

We next sought to examine if any of the sample-specific events could be attributed to Cas9 activity. We searched for gRNA-homologue sequences within 100 bp flanking regions of the detected indels and genomic rearrangements, along both directions and orientations. We identified only a single site in the intergenic region of chromosome 5 that shared >15 bp (>75%) sequence similarity with the gRNA (Fig. 2a; chr5: 7,924,778–7,924,799, hereafter termed as Chr5_OT). The remaining indels found in clones TAZ1, 2 and 4 were mostly located in repetitive regions and no sites within 100 bp flanking regions share >75% sequence similarity (>15 out of 19 bp) with gRNA sequence. Such mutations probably derive from spontaneous mutations during hiPSCs *in vitro* culture as reported before^18^,^19^.

Interestingly, at the Chr5_OT indel site the PGP1 hiPSC cell line used for this experiment harbours a heterozygous G/C SNV (dbSNP rs72716547). In reference to the hg19 genome, the *TAZ*-targeting gRNA has three mismatches to the Chr5_OT site at positions 11, 15 and 19 upstream of the PAM sequence. However, in the PGP1 genome a SNV ‘corrects’ the mismatch at position 11 and thus one of the Chr5_OT alleles has only two mismatches to the gRNA (Fig. 2a; Chr5_OT_var_). While Cas9 did not affect the reference Chr5_OT_ref_ allele in either of the two *TAZ*-knockout clones, the variant allele Chr5_OT_var_ showed small deletions in both clones (Fig. 2a). The allele-specific manner of Cas9 editing suggests strong mismatch discrimination and a very high specificity of the CRISPR/Cas9 system.

### Deep sequencing confirms high mismatch sensitivity of Cas9

Whole-genome sequencing revealed minimal off-target effects of Cas9 in modified hiPSCs. Observations made in only a few clones, however, did not yield quantitative results. To quantify potential off-target effects, we performed deep sequencing of predicted off-targets in a population of cells subjected to Cas9 induction and gRNA transfection. We identified 32 loci in the reference human genome (hg19/GRCh37) with no more than three mismatches to the gRNA-target site^20^ (Supplementary Table 1). Among those sites, the Chr5_OT_var_ allele was the only site with no more than two mismatches to the gRNA. We transfected PGP1-hiPSC-Cas9 cells with the *TAZ*-targeting gRNA and successfully deep sequenced 31 out of 32 potential off-target sites along with the *TAZ* target site (Supplementary Table 2). At the *TAZ* target site, 54% of DNA-sequencing reads exhibited short indels suggestive of Cas9 activity. Of the 31 potential off-target sites, the Chr5_OT off-target site was the only locus that exhibited a substantial-indel frequency (18.9%, Fig. 2b), while other off-target sites showed an average-indel frequency of ~0.15% (Fig. 2b, Table 2). Consistent with our observations from whole-genome sequencing data, we found strong allele-specific targeting at Chr5_OT: The variant allele Chr5_OT_var_ with two mismatches to gRNA showed a 36.7% indel frequency, while Chr5_OT_ref_ with three mismatches only showed a 1% indel frequency, similar to the efficiency observed at other off-target sites (Fig. 2c). The high specificity of Cas9 editing was further supported by the observation that more than two mismatches between gRNAs and target site resulted in significant decrease of the cutting efficiency (Fig. 2d). Together, our results indicate minimal off-target effects of Cas9, suggesting it is feasible to screen for hiPSC clones not harbouring off-target effects.

### *In silico* analysis reveals that SNVs impact Cas9 specificity

**Figure 3:**
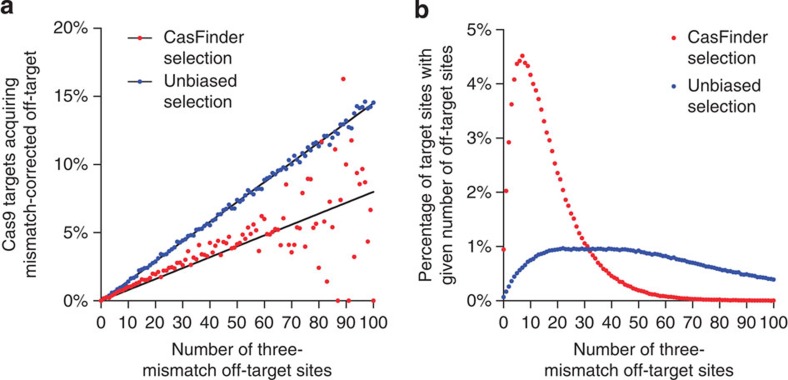
*In silico* analyses estimating the rates at which genome-variations reduce the specificity of Cas9. (**a**) Linear relations govern reduction of Cas9 specificity due to genome variations. We analyzed the effect of PGP1 SNVs determined from whole-genome sequencing on all unique Cas9 target sites in the human exome (‘unbiased’), and in all exomic-sites identified by a Cas9 site-selection algorithm (‘CasFinder’). Shown is the fraction of sites for which SNVs converted a three-mismatch off-target to a two-mismatch off-target, where sites are binned by number of three-mismatch off-target sites. Dots represent fractions of sites acquiring two-mismatch off-targets for each bin; lines represent linear regressions in the range of 0–100 off-targets assuming zero y- intercepts. The smaller regression slope for CasFinder (0.08%, *R*^2^=0.4795) versus that for the unbiased site selection (0.15%, *R*^2^=0.9975) indicates that Cas9 site-selection algorithms can reduce but do not eliminate SNV-generated off-targets. (**b**) Distribution of three-mismatch off-target counts of the Cas9 targets sites in (**a**). CasFinder-selected sites show a distribution of three-mismatch off-target counts that is highly concentrated to lower values (16.29±13.84, mean±st.dev) compared with the unbiased site selection (46.83±25.76; *P*=0, 2-sided *Z*-test). However, the low CasFinder regression rate in (**a**) suggests that the reduction of SNV-generated off-target rates by site-selection algorithms does not depend solely on filtering out sites with high numbers of off-targets. (See Supplementary Notes 1 and 2 for details).

To evaluate the scope of impact of SNVs on Cas9 specificity, we conducted an *in silico* analysis to calculate the likelihood of having high probable off-target sites due to SNVs in a given human genome. We took advantage of the availability of a comprehensive list of genome variations for the PGP1 cell line^21^, and tailored our analyses to the specificity characteristics of the *TAZ*-targeting site (Supplementary Note 1). First, the probability of finding a SNV-generated off-target should, in principle, increase linearly with the number of potential off-targets. We verified this by finding all three-mismatch off-targets for all unique Cas9 target sites (1,922,668 sites), computing the fraction that are converted to two-mismatch off-target sites by SNVs in PGP1 genome, and finding a very strict linear relationship (regression slope=0.145, *R*^2^=0.9975; Fig. 3a and Supplementary Note 2). However, Cas9 target sites are usually chosen using algorithms that purport to select sites with high specificity, which could potentially bias selection towards targets with less potential for SNP-mediated off-target generation. To gauge the impact of SNVs on targets chosen in this manner, we analyzed a database of 927,104 specificity-checked Cas9 target sites in the human exome that had previously been generated by the CasFinder algorithm^22^ and identified for each target site all three-mismatch off-target sites genome-wide. For target sites selected this way we found a probability of 1.54% that SNVs in the PGP1 genome lead to conversion of three-mismatch off-target sites to two-mismatch sites. This probability is lower than the 2.34% rate predicted by regression for the unbiased target-site selection (Supplementary Note 1). Indeed, plotting the rates of conversion of CasFinder sites by numbers of three-mismatch off-targets reveals a lower regression rate of 0.08% than the 0.145% of the unbiased sites (Fig. 3a). As expected, the CasFinder-selected sites show a distribution of three-mismatch off-target counts that is concentrated to lower values. (Fig. 3b; 16.17±12.56 versus 46.83±25.76; *P*=0, two-sided *Z*-test; Supplementary Note 1). Our results suggest that Cas9 site-selection software can reduce, but not eliminate, the occurrence of potential off-targets generated by SNVs in a genome.

As different ethnic groups have different SNV rates^23^ and the PGP1 cell line is derived from an individual of European descent, for comparison, we conducted an additional regression analysis on the genome of an individual of African descent (NA19240). We observed a larger regression slope (regression slope=0.182, *R*^2^=0.9973, Supplementary Fig. 7), consistent with the elevated SNV rate of African populations^23^. The overall frequency of two-mismatch off-target acquisition due to SNVs of the CasFinder-selected sites was 1.96% in this genome. Thus, the rate at which SNVs can generate potential off-targets undetected by Cas9 site-selection software is ~1.5–2.0%, depending on genome ancestry.

## Discussion

Here we have presented an exemplary case of genome-wide characterisation of Cas9 specificity. Through whole-genome sequencing of single-cell derived clones, we verified that there are no large-scale genomic structural rearrangements or copy-number alterations. The comparable rates of *de novo* indel observed in the *TAZ*-knockout clones and in the control clones also suggest that Cas9 editing does not induce mutagenesis on a large scale. The sole high-efficiency off-target site identified by whole-genome sequencing further indicates high mismatch sensitivity of Cas9 editing. These observations are further confirmed through deep sequencing of potential off-targets sites. The specificity of the CRISPR/Cas9 system thus matches the specificity of previously described zinc-finger nucleases^19^ and TALENs^24^,^25^ on the whole-genome scale. Our results, consistent with recent whole-genome analysis studies of Cas9 specificity^12^,^13^, have demonstrated that it is feasible to obtain *ex vivo* Cas9-modified hiPSC clones with minimal off-target mutations. We look forward to applications in other cell types and testing other systems with higher specificity requiring two gRNAs per cleavage^11^.

However, our discovery of an unexpected off-target site highlights the necessity for sample-specific gRNA design, especially in therapeutic applications of CRISPR/Cas9. Here we demonstrate how a single germline SNV can create a recurrent off-target site that can be missed by *in silico* predictions based on a reference genome sequence. Our large-scale *in silico* analysis quantifies this observation and reveals a considerable impact of SNVs on Cas9 specificity. We estimate the lower boundary to be ~1.5% for exomic-sites chosen by Cas9 site-selection algorithms on an European genome, but specifying an upper boundary is more difficult. A maximum frequency of off-target generation would be obtained in African genome, NA19240, for the complete set of unbiased exomic sites, but this set includes sites in repetitive sequence that would rarely be targeted in research or clinically. To set a practical upper boundary, we restrict attention to unbiased exomic sites with up to 100 three-mismatch off-targets as analyzed above, where this rate was ~8.5%. However, we note that sites being selected for clinical purposes will most often be chosen using Cas9 site-selection algorithms, and that the range of SNV-generated off-targets for such sites is ~1.5–2.0% site (considering European and African genomes, respectively). Moreover, Cas9 will not likely be as active at such off-targets as at the programmed target: If we extrapolate from our finding that Cas9 activity at site Chr5_OT was ~33% of that at the TAZ target, and assume that SNV-generated off-targets will (like Chr5_OT) mainly be heterozygous, the chance that Cas9 will generate a mutation at such an off-target for a randomly-picked algorithm-selected target will be ~0.5–0.66% relative to the target. This does not take into account the possibilities that the SNVs may be in PAM-distal positions where they do not increase Cas9 activity, and that even at off-target loci where Cas9 activity does increase, the locus may be unimportant to cell function or clinical utility. As these effects are difficult to quantify, we therefore simply estimate conservatively that for most Cas9 sites chosen for clinical purposes, the frequency of a functionally-important mutation arising in an off-target created by an SNV is, on average, <1% of the rate at the target site. However, we note that these estimates are based on the measured activities and specificity characteristics of our experimentally studied TAZ gRNA site, which proved active at two-mismatch off-targets but not at three-mismatch off-targets. While we expect these characteristics to be representative of a large class of gRNAs, they may not be shared by others for which estimates based on different characteristics would be required. Considerations of general estimation aside, precise analysis in particular cases would require a whole-genome variation file for the genome of interest. Taken together, our results suggest that whole-genome sequencing and sample-specific gRNA design are critical for achieving high specificity of Cas9 applications.

## Methods

### Genome editing of hiPSCs

PGP1 iPSCs were acquired from Personal Genome Project^15^. Cells were maintained on Matrigel (BD Biosciences)-coated plates in mTeSR1 medium (Stemcell Technologies) at 37 °C and 5% CO_2_ in a humidified incubator. Cultures were passaged every 5–7 days with TrypLE Express (Invitrogen). Genome editing on iPSCs using PiggyBac-inserted Cas9 was described before^16^. Briefly, PiggyBac carrying inducible Cas9 was integrated into the genome aided by transposase to generate Cas9-PGP1 iPSCs. After establishing the cell line, we transfected the cells with gRNA constructs through nucleofection and added 1 μg ml^−1^ doxycycline to the media to induce Cas9 expression. Single cells were expanded into clones and on-target effects were validated through Sanger sequencing following the previously described protocol^26^.

### DNA library preparation and sequencing analysis

Genomic DNA was extracted with DNeasy Blood & Tissue Kit (Qiagen) following manufacturer's instructions and subjected to standard whole-genome DNA-library preparation; whole-exome DNA libraries were captured on the Agilent Sure-Select Human All Exome v2.0 hybrid selection array. Each whole-genome DNA library was sequenced on the HiSeq platform (Illumina) to a mean coverage of 15x and each whole-exome library was sequenced to a mean coverage of 80x. Sequencing reads were aligned to the human reference genome (hg19/GRCh37) using Burrows–Wheeler Aligner (http://bio-bwa.sourceforge.net/)^27^ in the ‘mem’ mode (‘bwa mem’) with default parameters. Reads corresponding to PCR duplicates were removed by MarkDuplicates from PICARD (http://picard.sourceforge.net/), followed by base-quality recalibration and indel realignment by GATK^28^ (http://www.broadinstitute.org/gatk/). SNVs and indels from whole-genome data were detected by HaplotypeCaller from GATK jointly from all three samples, followed by variant-score recalibration and filtering with HapMap and 1000-genome training data following the best practices (http://www.broadinstitute.org/gatk/guide/best-practices). Variants were detected from whole-exome sequencing data by UnifiedGenotyper from GATK and further filtered on exon targets and individual sample genotypes were filtered on a genotype quality score of 50. Copy-number alterations were detected based on sequence-read depths by SegSeq^29^; chromosomal rearrangements were detected from clusters of discordantly mapped reads with inferred insert size longer than 1 kb or mapped to different chromosomes. The observation of distinct frameshift mutations due to Cas9-nuclease activity at the target sites in different knockout clones suggested that even site-direct DNA double-strand breaks do not result in identical mutations. We therefore assumed that off-target hits by Cas9 will not independently generate the same *de novo* mutations in different single-cell derived clones and only considered mutations that occur in each individual sample. Discarding all variants shared by two or more samples not only enabled us to exclude pre-existing mutations but also provided an efficient filter against noises and artifacts in the bioinformatic analysis. For *de novo* rearrangement detection, we required true variant to be supported by at least four discordant reads (including multi-part alignments or ‘split reads’). Each unique variant (indel and structural variants) was visually confirmed using IGV^30^.

### Site-specific deep sequencing

Cas9-PGP1 iPSCs were nucleofected using P3 Primary Cell 4D-Nucleofector X Kit (Lonza). A total of 5 × 10^5^ cells were harvested using TrypLE Express (Invitrogen) and resuspended in 20 μl nucleofection mixture containing 16.4 μl of P3 Nucleofector solution, 3.6 μl supplement, 1 μg of gRNA construct (and 1 μg Cas9 construct for transient transfection of Cas9). The nucleofection reactions were then conducted using the CB150 program. Thereafter the cells were plated on Matrigel-coated plates in mTeSR1 medium supplemented with ROCK inhibitor (Calbiochem Y-27632) and 1 μg ml^−1^ DOX. After 48 h the cells were harvested and the genomic DNA was extracted using ZyGEM prepGEM extraction kit. Primers used in the deep sequencing can be found in Supplementary Table 3. The libraries were purified with QIAquick PCR Purification Kit (Qiagen) and sequenced on the MiSeq platform. Cas9 activity was measured through indel detection and relative Cas9 cutting efficiencies were calculated by normalising the off-target cutting efficiency to on-target efficiency.

## Author contributions

L.Y. and G.C. conceived the study; L.Y. and G.W. performed the iPSCs editing using CRISPR; X.C., Y.Z. and J.-B.F. performed the WGS and preliminary data analysis; D.G. and C.-Z.Z. performed the WGS analysis and validation. J.A. and R.C. performed regression and stimulation on how SNVs impact specificity. D.G. and L.Y. performed the deep sequencing experiments and analysis. D.G., L.Y. and C.-Z.Z. wrote the manuscript with the help of other co-authors. L.Y., D.G., J.A., and R.C. were supervised by G.C.

## Additional information

**How to cite this article:** Yang, L. *et al*. Targeted and genome-wide sequencing reveal single nucleotide variations impacting specificity of Cas9 in human stem cells. *Nat. Commun.* 5:5507 doi: 10.1038/ncomms6507 (2014).

**Accession codes:** Sequencing data has been deposited in BioProject under accession number PRJNA259786.

## Supplementary Material

Supplementary Information Supplementary Figures 1-7, Supplementary Tables 1-3, Supplementary Notes 1-2 and Supplementary References

## Figures and Tables

**Table 1:** Cas9 activity does not increase the rate of genomic rearrangements above background.

	**Genomic rearrangements**			
	**Deletions**	**Duplications**	**Inversions**	**CNV>100 kb**
Control clone	2	0	1	0
TAZ1 clone	1	1	2	0
TAZ2 clone	1	0	0	0

Number of *de novo* genomic rearrangements in the control clone and TAZ1 and TAZ2 clones as detected by whole-genome sequencing analysis.

**Table 2:** Potential off-target sites with one to three mismatches to gRNA and their observed-targeting efficiencies.

**Target and off-target indel frequencies**		
**Number of mismatches**	**Number of genomic sites**	**Cas9 targeting efficiency**
0	1	53.9%
1	0	—
2	0→1	36.7%
3	32	~0.15% per site

The variant allele of Chr5_OT represents the only site with only two mismatches to the gRNA and Cas9 target efficiency at this site was observed to be 36.7%, comparable with that of the target site (53.9%); the remaining sites with three mismatches were targeted by Cas9 with an average efficiency of ~0.15% (Supplementary Table 2) as measured by deep sequencing.
