# Supplementary material for: Targeted and genome-wide sequencing reveal single nucleotide variations impacting specificity of Cas9 in human stem cells
Source: Nat Commun. 2014 Nov 26;5:5507. doi: 10.1038/ncomms6507 (PMC4352754; doi:10.1038/ncomms6507)
Supplement: Supplementary Information — Supplementary Figures 1-7, Supplementary Tables 1-3, Supplementary Notes 1-2 and Supplementary References [file ncomms6507-s1.pdf]

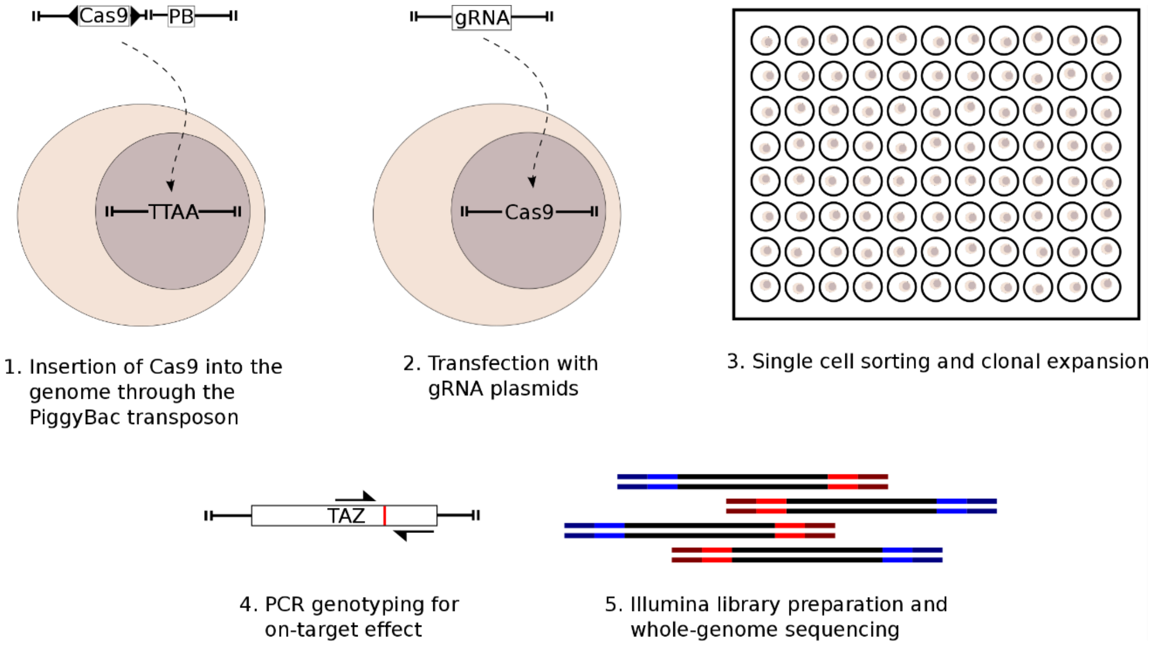

Supplementary Figure 1| Experimental design

Cas9 was inserted in the genome of hiPSCs through *PiggyBac* transposon. Cells were transfected with *TAZ*-targeting gRNA and Cas9 induced by doxycycline. Single cells were sorted and expanded to colonies. Single-cell derived colonies were genotyped for on-target effect in *TAZ* gene. Whole-genome Illumina libraries were generated from cloned cells and sequenced. The control sample was transfected with a gRNA that has a minimum of 4 mismatches against the human genome reference.

a

b

c

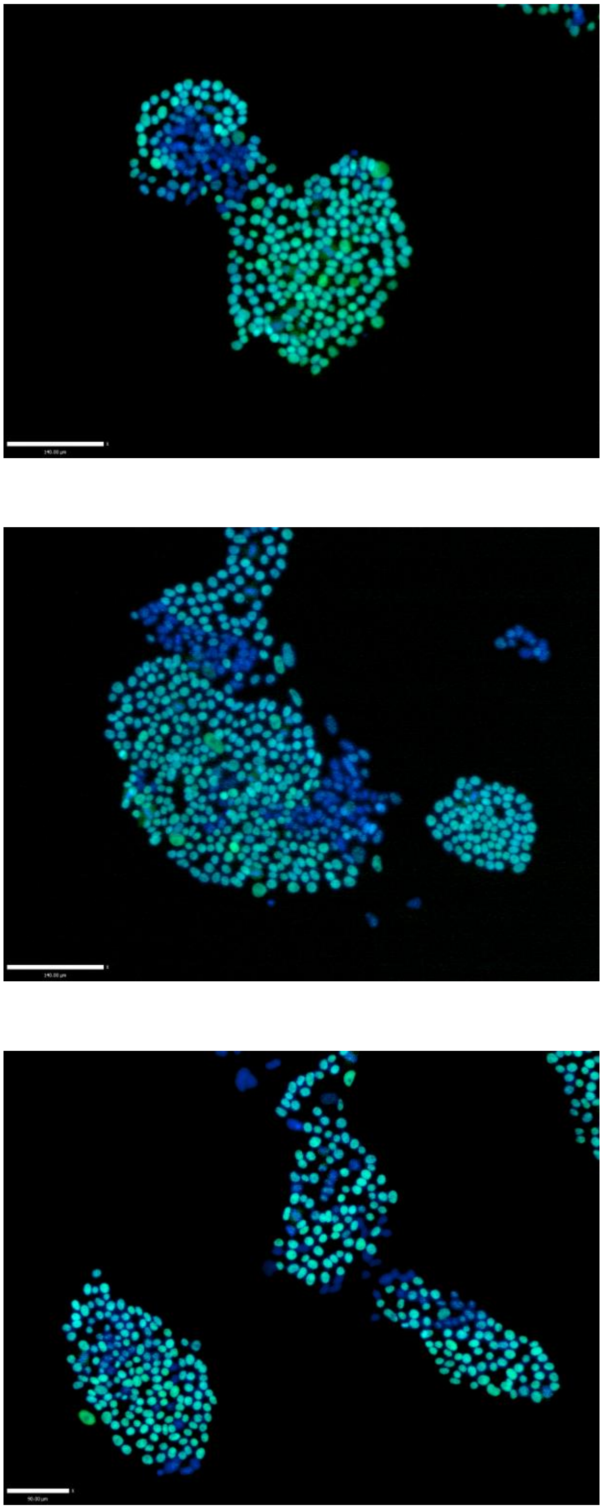

Supplementary Figure 2| OCT4 immunostaining of control clone (a) and clones TAZ3 (b) and TAZ4 (c) verified pluripotent state of hiPSCs

Scale bar =140 *µm*. The methodology has been described before by Wang *et al.* [1].

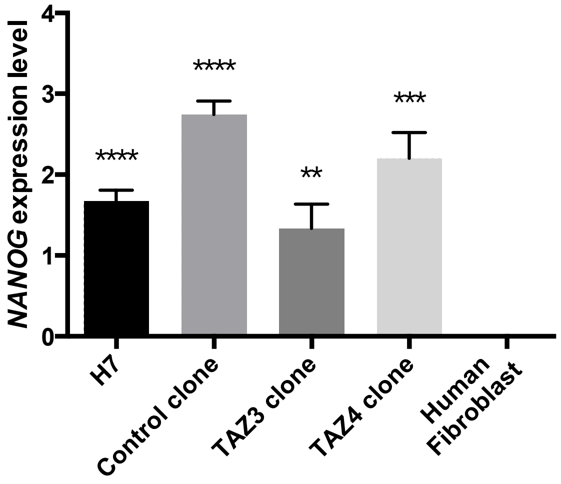

Supplementary Figure 3| *NANOG* expression levels in control clone and clones TAZ3 and TAZ4 validated the pluripotent state of hiPSCs
Genome modified PGP1 hiPSC clones showed significant higher *NANOG* expression levels compared to human fibroblasts, comparable to that of hES cell line H7. (N=3; S.D.; *t*-test: ** p<0.005, *** p<0.0005) measured by RT-QPCR. The methodology of RT-QPCR has been described before by Wang *et al.* [1].

	# of Indels
Control	0
TAZ3	0
TAZ4	1

Supplementary Figure 4| Indel number detected in the exoms of control clone and clones TAZ3 and TAZ4

Samples were compared to the control clone to filter out preexisting mutations. We do not observe any sequence similarity to *TAZ* on-target site in the flanking regions (±100bp) of 2bp deletion detected in clone TAZ4 (chr2: 24260743; CGA→C).

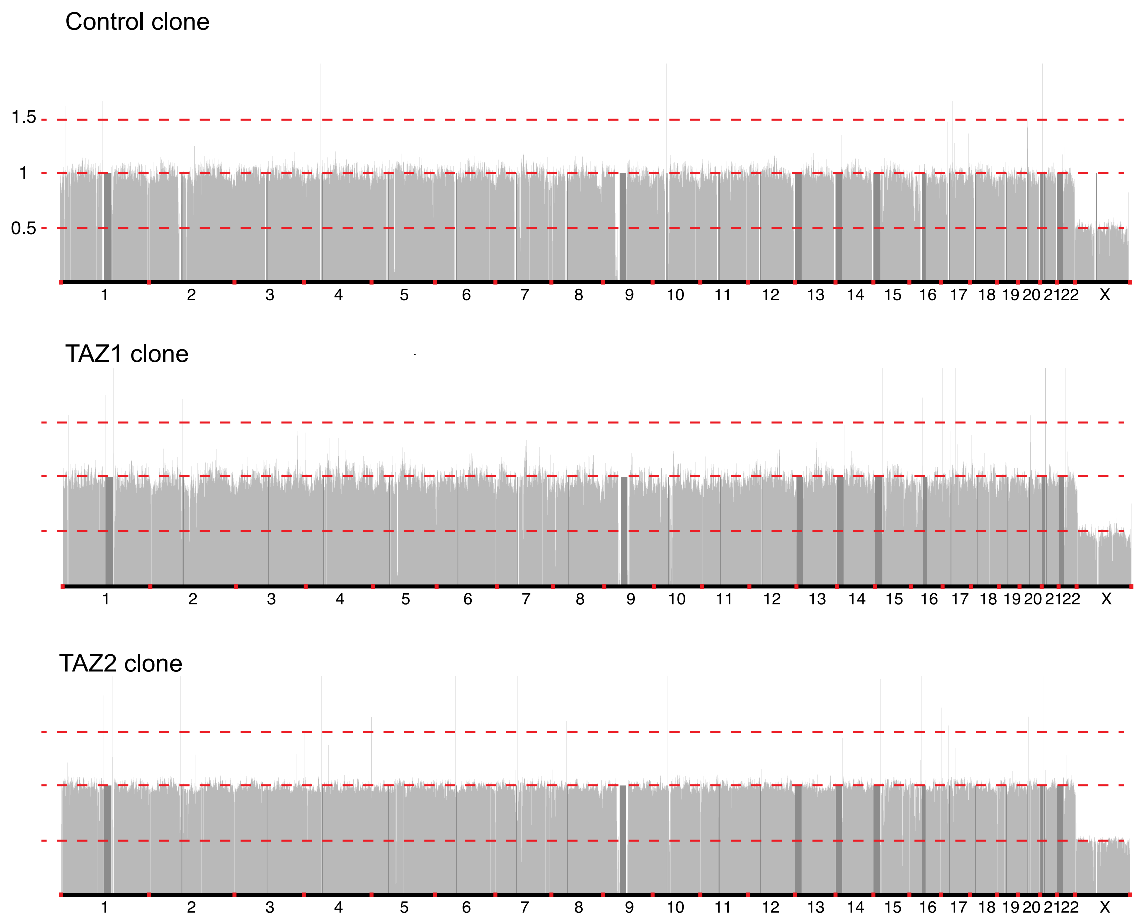

Supplementary Figure 5| No copy number variations were observed in clones TAZ1 and TAZ2

Sequencing coverage is shown in 500 kb bins after GC bias correction.

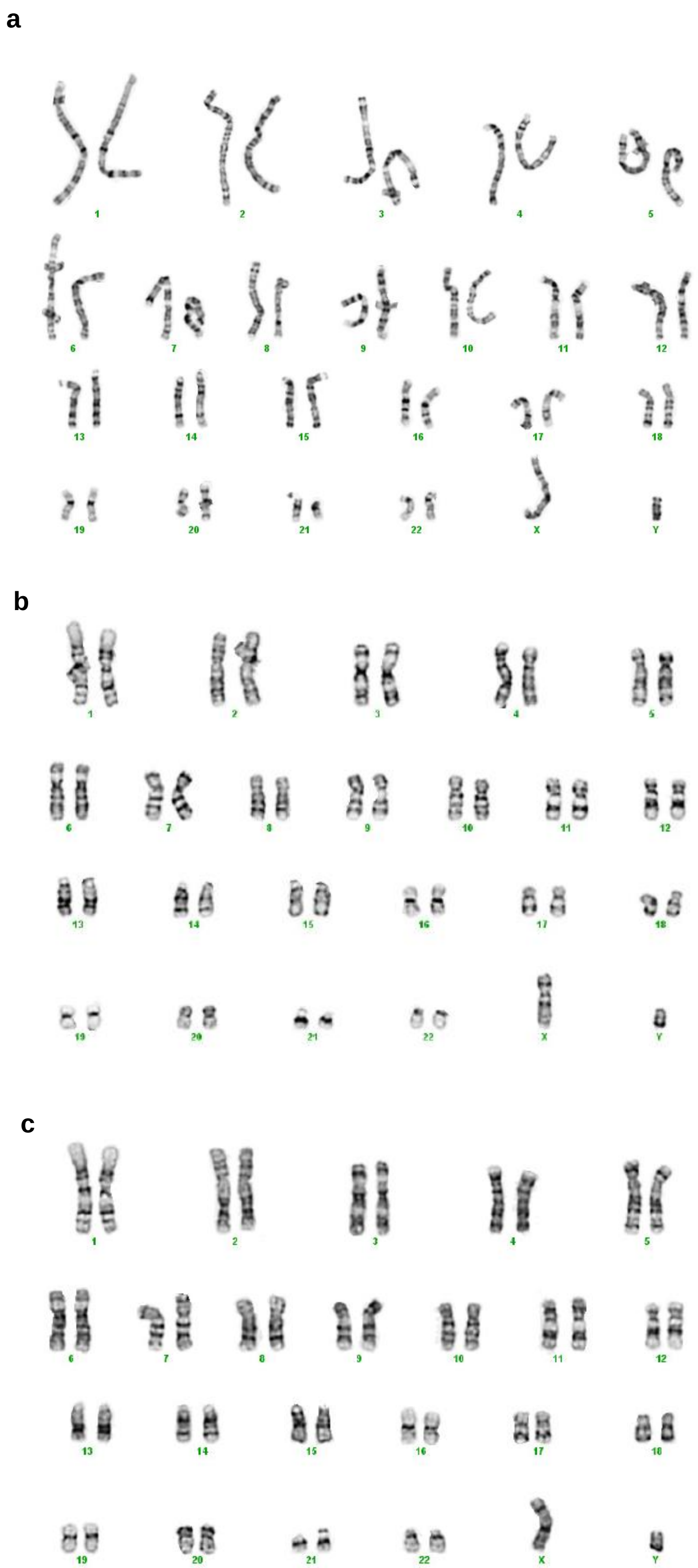

Supplementary Figure 6| Karyotypes of control clone (a) and clones TAZ3 (b) and TAZ4 (c) verified genomic integrity

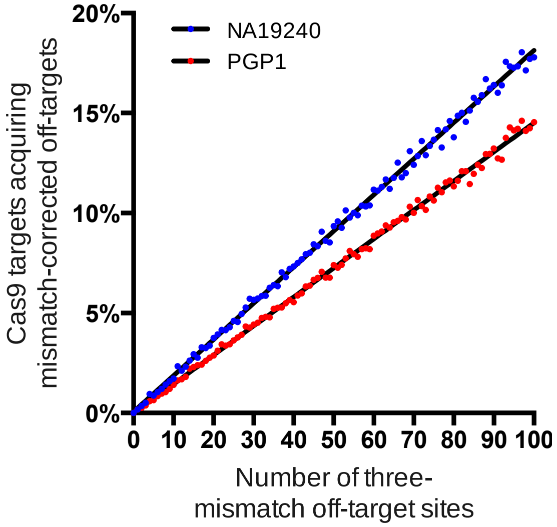

Supplementary Figure 7| Effect of ethnical background on the incidence of mismatch-corrected off-target sites

Strict linear relations govern reduction of Cas9 specificity due to genome variation. All Cas9 target sites with no off-targets closer than three mismatches were identified in the human exome. Shown is the fraction of target sites for which SNVs, identified from whole-genome sequencing, converted a three-mismatch off-target to a two-mismatch off-target site, where target sites are binned by number of three-mismatch off-target sites. *TAZ*-knockout experiments described in the main text were conducted using the PGP1 cell line, which is of European ancestry. For comparison, this analysis was repeated using an SNV list obtained from an individual of African ancestry (NA19240). As expected from the elevated variation rate in African genomes, the NA19240 genome exhibits a higher rate of loss of Cas9 specificity as seen in the larger regression slope.

1.	AAGCTCCACCTTGGGAACTTGG	17.	AAGCTCAAGCATGTGGATTTGG
2.	AACTTCAACCATGGGAACTAGG	18.	AAGCTCTACCCTGGGGACCTGG
3.	AAGTGCAACCATGAGGACTTGG	19.	AAGCCCAACCTTGTGGACTAGG
4.	AATCCCAACCATGGGGCCTGGG	20.	AAGCCCAACCAAGGGCACTGGG
5.	GAGCTCAACCCTGGAGACTGGG	21.	AAGCAGAACCATCGGGACTTGG
6.	AGGCTCAGCCATGGGGGCTGGG	22.	AAGCTCAACCATGGGCTGTGGG
7.	AGGCTCAGCCATGGGGGCTGGG	23.	AAGCTCAGCCATAGGGGCTGGG
8.	AGGCTCAGCCATGGGGGCTGGG	24.	AAGTCCAAACATGGGGACTTGG
9.	AGGCTCAGCCATGGGGGCTGGG	25.	AAGCTCAACCATGGGGCTGTGG
10	AGGCTCAGCCATGGGGGCTGGG	26.	GAGCTCAACCATGAGTACTTGG
11.	AGGCTCAGCCATGGGGGCTGGG	27.	AAGCCCCACCTTGGGGACTAGG
12.	AGGCTCAGCCATGGGGGCTGGG	28.	CAGCACAAGCATGGGGACTTGG
13.	AAGCTCAGCCATGGAGGCTAGG	29.	AACCTAAAGCATGGGGACTGGG
14.	AAGCTCAGCCATGGAGGCTGGG	30.	AAGCTCCACCATGGGCCCTGGG
15.	AACCTCAACCATAGGAACTTGG	31.	ACGCCCAACCATGGGAACTTGG
16.	GAGCTCAACCTTGGGCACTAGG	32.	AAGCTCAAGAATGGGGAATGGG

Supplementary Table 1| List of all potential off-target sites of *TAZ*-targeting gRNA with three mismatches

Of note, all sites have at least one mismatch within the 12 bases upstream of PAM. The high frequency off-target site Chr5_OT revealed by WGS is highlighted in gray.

Off-target site	Total reads #	Indel reads #	Indel frequency
1	352679	267	0.08%
2	2583	4	0.15%
3	270107	147	0.05%
4	3651	16	0.44%
5	434	4	0.92%
6	110549	663	0.60%
7	48843	204	0.42%
8	4243	20	0.47%
9	2190	11	0.50%
10	182504	744	0.41%
11	3404	3	0.09%
12	150138	579	0.39%
13	4201	5	0.12%
14	10020	52	0.52%
15	1454	0	0.00%
16	268205	298	0.11%
17	2012	5	0.25%
18	1022	0	0.00%
19	767	0	0.00%
20	193618	272	0.14%
21	26706	28	0.10%
22	353484	880	0.25%
23	4438	4	0.91%
24	386036	1145	0.30%
25	454280	352	0.08%
26	2012	13	0.65%
27	4124	29	0.70%
28 (Chr. 5)	18682	3533	18.91%
29	412622	463	0.11%
30	2313	12	0.52%
31	5185	16	0.31%

Site	Total reads #	Indel reads #	Indel frequency
TAZ-target site (negative control)	5353	9	0.17%
TAZ-target site	412622	223898	54.26%

Supplementary Table 2| Indel frequencies detected at the on- and off-target sites using deep sequencing

The sequences of off-target site are listed in Table S1. The negative control data was obtained from deep sequencing of same cell line without Cas9 induction and gRNA transfection. The frequency of 0.17% observed in the negative control seems to be largely contributed by sequencing errors [2]. Thus, the indel frequencies observed for all off-target are adjusted by subtracting the background indel rate of 0.17%. Based on this, we estimated the average off-target effect at the 32 three-mismatch sites to be ~ 0.15%. Of note, it was repeatedly demonstrated that the level of Cas9 activity at potential off-target sites is reversely correlated to the number of mismatches and Cas9 has been shown to not cut sites with >3 mismatches [3,4]. Therefore we do not expect significant Cas9 effects at off-target sites with >3 mismatches.

TAZ_F	5'-acactctttccctacacgacgctcttccgatctCCCCGAGAATGGTTACTGAT-3'
1_F	5'-acactctttccctacacgacgctcttccgatctTCCTTTGCAGACTTCACTCAA-3'
2_F	5'-acactctttccctacacgacgctcttccgatctCAGGGCAGCTCTCTTCTACG-3'
3_F	5'-acactctttccctacacgacgctcttccgatctCAAAACTTTGGGAAATGTGAAT-3'
4_F	5'-acactctttccctacacgacgctcttccgatctCAGGAGCCAGGGGTGTTT-3'
5_F	5'-acactctttccctacacgacgctcttccgatctGCTGGAAGACTTTTGCACCT-3'
6_F	5'-acactctttccctacacgacgctcttccgatctGGAGCACCCATGTCTACCA-3'
7_F	5'-acactctttccctacacgacgctcttccgatctTCCAGAGACCACCCACATCT-3'
8_F	5'-acactctttccctacacgacgctcttccgatctAGGTGCAGAGCGTCCTTG-3'
9_F	5'-acactctttccctacacgacgctcttccgatctCAGGGAGCACCCATGTCTA-3'
10_F	5'-acactctttccctacacgacgctcttccgatctCAGGTGCAGAGCGTCCTT-3'
11_F	5'-acactctttccctacacgacgctcttccgatctAGGTGCAGAGCGTCCTTG-3'
12_F	5'-acactctttccctacacgacgctcttccgatctCAGGTGCAGAGCGTCCTT-3'
13_F	5'-acactctttccctacacgacgctcttccgatctCTGTTCCAAGAGCCCACAAG-3'
14_F	5'-acactctttccctacacgacgctcttccgatctCGACTCCTAGTTGCAGGTACA-3'
15_F	5'-acactctttccctacacgacgctcttccgatctAGCCTTAGCAAAAGCGAAAGT-3'
16_F	5'-acactctttccctacacgacgctcttccgatctTTCTGCATAGAGGGAAACAGG-3'
17_F	5'-acactctttccctacacgacgctcttccgatctCAGAGTGGGTAAAATGGAGAAT-3'
18_F	5'-acactctttccctacacgacgctcttccgatctGGCAGAAGACTTGAACTTCTCAG-3'
19_F	5'-acactctttccctacacgacgctcttccgatctAGAAAGCCTCTGAGGACCTG-3'
20_F	5'-acactctttccctacacgacgctcttccgatctTGAGGAGAACAATGATACTGGTTT-3'
21_F	5'-acactctttccctacacgacgctcttccgatctAGGTGACAGGAGTAAGGACCTG-3'
22_F	5'-acactctttccctacacgacgctcttccgatctTTAACAGCAGAAGGACAGATGG-3'
23_F	5'-acactctttccctacacgacgctcttccgatctGGAGTTTCCCCGTTCACC-3'
24_F	5'-acactctttccctacacgacgctcttccgatctTAACATAAAACTTTCAGATGTAGCTGT-3'
25_F	5'-acactctttccctacacgacgctcttccgatctGATACACACATTCAGAGCATCCTG-3'
26_F	5'-acactctttccctacacgacgctcttccgatctAGGGGCCCAAAACATTTAAC-3'
27_F	5'-acactctttccctacacgacgctcttccgatctCAGGGGACTCATCTTCCAAT-3'
28_F	5'-acactctttccctacacgacgctcttccgatctCACCACTGCAGGCACAGT-3'
29_F	5'-acactctttccctacacgacgctcttccgatctTTGTGGAACTCGTGCAAGC-3'
30_F	5'-acactctttccctacacgacgctcttccgatctGGTACACCCATTCCTCTTCC-3'
31_F	5'-acactctttccctacacgacgctcttccgatctCTGCCACTGGTCAGCTATGA-3'
32_F	5'-acactctttccctacacgacgctcttccgatctCACACTCCTGCAGCCAGA-3'
TAZ_R	5'-gtgactggagttcagacgtgtgctcttccgatctCCATCCCGCTCATACTGG-3'
1_R	5'-gtgactggagttcagacgtgtgctcttccgatctTGATACATGAAGAAAGAGCATCC-3'
2_R	5'-gtgactggagttcagacgtgtgctcttccgatctTTCCCCAGACTTTGCTCTTT-3'
3_R	5'-gtgactggagttcagacgtgtgctcttccgatctGACCCTTTTCACTGCAAACC-3'
4_R	5'-gtgactggagttcagacgtgtgctcttccgatctGAAACGAGCTCCGCAAGC-3'
5_R	5'-gtgactggagttcagacgtgtgctcttccgatctGCTGGCAGAGCTCAGGTTAT-3'
6_R	5'-gtgactggagttcagacgtgtgctcttccgatctCCGGATGCAGGTACCAAG-3'
7_R	5'-gtgactggagttcagacgtgtgctcttccgatctAGGTGCAGAGCGTCCTTG-3'
8_R	5'-gtgactggagttcagacgtgtgctcttccgatctCAGGGAGCACCCATGTCTAC-3'
9_R	5'-gtgactggagttcagacgtgtgctcttccgatctCCGGATGCAGGTACCAAG-3'
10_R	5'-gtgactggagttcagacgtgtgctcttccgatctTCCAGAGACCACCCACATCT-3'

11_R	5'-gtgactggagttcagacgtgtgctcttccgatctCAGGGAGCACCCATGTCTAC-3'
12_R	5'-gtgactggagttcagacgtgtgctcttccgatctTCCAGAGACCACCCACATCT-3'
13_R	5'-gtgactggagttcagacgtgtgctcttccgatctCGCCTAGCTGCAGGTACAGA-3'
14_R	5'-gtgactggagttcagacgtgtgctcttccgatctCAGAGAGAGCCCATGTCTGC-3'
15_R	5'-gtgactggagttcagacgtgtgctcttccgatctCTCAGGCTTAGCTGGCTCAC-3'
16_R	5'-gtgactggagttcagacgtgtgctcttccgatctTGCGCTTCCTCTTTTAGACC-3'
17_R	5'-gtgactggagttcagacgtgtgctcttccgatctTATCACATCCCTGCCCAAAT-3'
18_R	5'-gtgactggagttcagacgtgtgctcttccgatctCCATTTCACTGATTGAGAGACTG-3'
19_R	5'-gtgactggagttcagacgtgtgctcttccgatctCCAAACAGGCTGGAGCTTAG-3'
20_R	5'-gtgactggagttcagacgtgtgctcttccgatctTGGAGCACATGACAGTGGTT-3'
21_R	5'-gtgactggagttcagacgtgtgctcttccgatctCTCAAAACTTAAAAAGCATTTGG-3'
22_R	5'-gtgactggagttcagacgtgtgctcttccgatctCACACTGCAGAGAGCTGGAA-3'
23_R	5'-gtgactggagttcagacgtgtgctcttccgatctGCAGGGCCCTTGATTGAG-3'
24_R	5'-gtgactggagttcagacgtgtgctcttccgatctAATACTTGGTAAATGTGCTGAATG-3'
25_R	5'-gtgactggagttcagacgtgtgctcttccgatctACAGATCTGAAAATTAAGTGTAATGC-3'
26_R	5'-gtgactggagttcagacgtgtgctcttccgatctCTGCAGGCCTTGATAAGACA-3'
27_R	5'-gtgactggagttcagacgtgtgctcttccgatctTTCTGCTAGATTTCTTGCTTCCTT-3'
28_R	5'-gtgactggagttcagacgtgtgctcttccgatctGTAGGGAAGTTGCCCAGCAC-3'
29_R	5'-gtgactggagttcagacgtgtgctcttccgatctAATCCCTAAACCAAAGATACCC-3'
30_R	5'-gtgactggagttcagacgtgtgctcttccgatctCGCCTGTGATTCCTTAGAGC-3'
31_R	5'-gtgactggagttcagacgtgtgctcttccgatctAAATGGGCTGATTGTGGATG-3'
32_R	5'-gtgactggagttcagacgtgtgctcttccgatctTGTGATGCTCACATCCACTCT-3'

Supplementary Table 3| Primer sequences with Illumina adaptors (lower case) used for deep sequencing

We followed the protocol as described before by Yang *et al.* [2].

Supplementary Note 1 | Impact of SNVs on targets chosen by a Cas9 site specificity algorithm

Cas9 targets are frequently selected using software that attempts to enforce specificity constraints, frequently by filtering out candidate targets for which there are similar sequences in a genome that have potential to function as Cas9 off-targets [5–9]. There is reason to expect that sites chosen by these algorithms will be less susceptible to acquisition of active off-targets though the presence of SNVs. As seen in Figure 3a of our article, if a target has *n* similar sequences in the genome at a given Hamming distance, the chance that a SNV will convert one of these sequences to one with a smaller Hamming distance (and therefore to a more likely active Cas9 off-target) is proportional to *n*. By their filtering, Cas9 specificity algorithms will likely identify targets with smaller values of *n* than targets chosen without the algorithms, and thus smaller probabilities of a site acquiring a closer off-target through a SNV. However, it is also possible that Cas9 site selection algorithms bias sites towards sequences with base compositions that have different propensities to harbor SNVs. We explored these hypotheses using CasFinder [9], an algorithm in use in our laboratory, as a representative Cas9 target selection algorithm.

We started with a whole human exome database of 927104 Cas9 target sites (896658 unique) previously generated using CasFinder [9]. Because CasFinder's site evaluation differs from the specificity conditions of the TAZ gene Cas9 target, these sites were re-evaluated using bowtie [10] to find a subset meeting comparable conditions and to identify potential Cas9 off-targets of sites in this subset. (We note that the TAZ site tested in the article was not chosen by CasFinder and, indeed, failed its specificity tests.) Specifically: (i) In a version of the method applied in ref. [11], we ran bowtie with the –a and –v 3 options to query for all 22bp sequences in the genome that differed from any of CasFinder sites by up to three places excluding consideration of the N position of the NGG PAM. This was effected by issuing four 22bp bowtie queries per CasFinder site comprising the first 19bp of the CasFinder 20bp targeting sequences plus each of the four PAM sequences AGG, CGG, GGG, and TGG. For brevity we refer to the sequences returned by bowtie for each of the four queries per site as 'hits' in the genome for that site. Nineteen bp targeting sequences were used in the queries because the TAZ gene gRNA only contained a 19bp vs. 20bp targeting sequence. The bowtie options and multiple queries ensured that all sequences in the genome with up to 3 mismatches from the 19bp targeting sequence and followed by either an AGG, CGG, GGG, or TGG would be returned as hits for each CasFinder site. Some hits could be returned by multiple queries, and such duplicates were ignored. The set of hits for each site thus includes the space of 3bp mismatch off-targets that could be changed to <3bp mismatch off-targets by a SNV, matching the conditions observed for the TAZ gene gRNA, but additional relevant hits could also be returned such as those in which mismatches were found in the GG positions of the PAM. (ii) We then identified those CasFinder sites that lacked 2 bp mismatch off-targets in the genome, matching the specificity characteristics of the TAZ gRNA site. To do this, we used custom Perl code to classify each hit for a site as either an "excluded off-target" or a "non-excluded off-target", excepting the hit corresponding to the exact match with the site, which was ignored in all follow-on processing. Hits were considered 'excluded' off-targets if they contained a mismatch from the GG of the NGG PAM, or if the total number of mismatches to the 19bp targeting sequence in the query was 3. All other hits were considered 'non-excluded'. The subset of 611589 sites for which all off-targets were "excluded" was considered the "accepted subset" that matched the specificity conditions of the TAZ gRNA, and this was used in subsequent processing. The hg19 human genome reference sequence downloaded from the UCSC Genome Browser web site [12] was used for both CasFinder and bowtie analysis.

Whole-genome genome sequence variation files in GFF format were prepared for the PGP1 and NA19240 genomes from data available from ref. [13] using data and software resources from ref. [14]. SNV and SUB variations were extracted from these files for further use, and indels and other entries were ignored. A second custom Perl program was then used to alter the sequences of all the hits for the accepted subset of CasFinder sites to incorporate the derived alleles of each SNV or SUB variation that had a non-empty intersection with the hit. For each such alteration, the program re-evaluated the altered

hit sequence according to the criteria for exclusion described above to see if the adjusted hit sequences remained excluded off-targets. Sites for which any excluded off-target became non-excluded were counted as instances where a genome variation could acquire a Cas9 off-target in the manner observed for the TAZ gRNA. These sites are denoted “converted” sites, and their rates are indicated in Figure 3a of the article. We found that 1.558% of all accepted CasFinder sites were converted by the PGP1 genome. For consistency with our analysis of exonic Cas9 sites that were not chosen by a Cas9 target selection algorithm (see Supplementary Note 2), we recalculated the conversion rate using only SNV variations, and only using excluded off-targets that had a total of 3 mismatches to the original site sequence (including PAM GG mismatches). This yielded a slightly reduced conversion rate of 1.541%. The same analyses were conducted with the NA19240 genome and yielded higher conversion rates of 1.981% and 1.961%, respectively, consistent with the elevated variation rate in genomes of African ancestry (see Supplementary Figure 7).

We then constructed a frequency distribution of counts of 3bp mismatch off-targets per sites for all accepted CasFinder sites, using the criteria of Supplementary Note 2 as noted above. This is depicted in Figure 3b in the article. Consistent with the hypothesis above, CasFinder-selected sites exhibit a distribution that is much more concentrated at lower values than the corresponding distribution constructed from the data of Supplementary Note 2. The mean ± st. dev. of the complete distribution is 16.29 ± 13.84; however, to be consistent with the analysis of the unbiased target set from Supplementary Note 2, we focused on the 611051 sites (99.9% of the full distribution) with 3bp mismatch off-target counts in the range 0-100, for which the mean ± st. dev. was 16.17 ± 12.56. A two-sided *Z*-test comparison of the mean number of off-targets for sites in in this range between the CasFinder and the unbiased sets yields $Z = 1141.4$ ($P = 0$, 2-sided, computed in Excel).

Finally, we computed the actual fractions of sites in each of off-target count bin that acquired 2bp mismatch off-targets for the PGP1 genome, and computed the regression in Figure 3a in Excel. The regression slope is .08%, much lower than the .1451% of the Cas9 sites of Supplementary Note 2. The R^2 of this regression is 0.4795, considerably lower than the .9975 of the unbiased Cas9 sites. Statistical noise due to the low numbers of sites with high numbers of off-targets likely accounts for much of this effect.

The fact that CasFinder dramatically reduces the number of potential off-target sequences per site compared to choosing sites without a selection algorithm is consistent with the hypothesis stated at in the introduction to this Supplementary Note that site selection algorithms can lower the probability of a site acquiring an off-target through a SNV simply by reducing the number similar sequences that could be converted by a SNV. However, the fact that CasFinder sites exhibit a lower regression slope compared to unbiased sites suggests that additional mechanisms may play a role in the reduction of SNV-mediated off-target acquisition, for off-target acquisition rates are lower even for sites with off-target counts in the range 0-30, in which the CasFinder set has a much larger fraction of sites than the unbiased set (Figure 3b). This is consistent with the second hypothesis mentioned in the introduction, that site selection algorithms such as CasFinder may chose sites with a sequence composition that has a lower propensity to harbor SNVs, for instance, by biasing site selection to sequences with lower CpG frequencies that have elevated frequencies of SNVs [15]. We have not explored these possibilities at this time.

Finally, we note that, independent of the effect of algorithm-imposed specificity checks, there are additional reasons to expect that the conversion rates found by these analyses underestimate actual off-target conversion rates. Specifically, the whole genome sequencing of PGP1 and NA19240 did not call 100% of these genomes, so the variation files for these genomes must also be considered incomplete. Also, as previously noted, indels in these genomes were not considered in the simulation.

Supplementary Note 2 | Impact of SNVs on targets using an unbiased set of gRNA sequences

For most applications, algorithms such as CasFinder would typically be employed to identify target sites with minimal predicted off-target effects. However, there are instances for which the required target site may have unavoidable off-target effects. As such, it would be important to understand the impact of single nucleotide variations in introducing additional off-targets across an unbiased set of Cas9 sites with varying levels of specificity.

First, using the RefSeq annotation of genes, the sequences of all coding exons were obtained from the RefFlat table in the UCSC Genome Browser database.[12] Next, custom python scripts were used to identify all unique, putative N19-NGG sites within these sequences which resulted in the identification of 1922668 unique sites. Subsequently, these sequences were aligned to the hg19 version of the human genome using the SeqMap algorithm [16] allowing for up to four mismatches and obtaining all possible alignments. Four mismatches were used in the alignment in order to filter the alignments for the wildcard base at position 20 (N before the GG). That is, if an off-target was called with four mismatches with one of the mismatches at position 20, it was considered an off-target with three mismatches. Thus, for each of the unique sites, the number of three mismatch off-target sites was determined. The distribution of numbers of such off-targets has a heavy right tail, and we focused mainly on the 1431909 sites with between 0 and 100 three mismatch off-target sites (74.5% of the full set). The distribution of sites with numbers of off-targets in this range is depicted in Figure 3b. The mean $\pm$ std. dev. of the number of three mismatch off-targets per site in this set was 46.84 $\pm$ 25.77 (compared to 124.31 $\pm$ 2399.57 for the full set). Using only single nucleotide variations from either PGP1 or NA19240, we then calculated the percentage of Cas9 sites that had at least one three mismatch off-target become a two mismatch off-target due to a single nucleotide variation in the respective genome. Using the 101 data points, a linear regression was performed using Microsoft Excel where a slope and R^2 value were determined. A very strong linear relationship was observed between the number of three mismatch off-targets and the percentage of Cas9 sites in a respective bin being affected by a single nucleotide variation. The slope of the linear relationship varies depending on the genome, likely due to the different rates of SNVs in individuals.

Supplementary References

1. Wang, G. *et al.* Modeling the mitochondrial cardiomyopathy of Barth syndrome with induced pluripotent stem cell and heart-on-chip technologies. *Nat. Med.* (2014). doi:10.1038/nm.3545

2. Yang, L. *et al.* Optimization of scarless human stem cell genome editing. *Nucleic Acids Res.* **41,** 9049–61 (2013).

3. Hsu, P. D. *et al.* DNA targeting specificity of RNA-guided Cas9 nucleases. *Nat. Biotechnol.* **31,** 827–32 (2013).

4. Fu, Y. *et al.* High-frequency off-target mutagenesis induced by CRISPR-Cas nucleases in human cells. *Nat. Biotechnol.* **31,** 822–6 (2013).

5. Biswas, A., Gagnon, J. N., Brouns, S. J. J., Fineran, P. C. & Brown, C. M. CRISPRTarget: bioinformatic prediction and analysis of crRNA targets. *RNA Biol.* **10,** 817–27 (2013).

6. Xiao, A. *et al.* CasOT: a genome-wide Cas9/gRNA off-target searching tool. *Bioinformatics* (2014). doi:10.1093/bioinformatics/btt764

7. Heigwer, F., Kerr, G. & Boutros, M. E-CRISP: fast CRISPR target site identification. *Nat. Methods* **11,** 122–3 (2014).

8. Montague, T. G., Cruz, J. M., Gagnon, J. A., Church, G. M. & Valen, E. CHOPCHOP: a CRISPR/Cas9 and TALEN web tool for genome editing. *Nucleic Acids Res.* **42,** W401–7 (2014).

9. Aach, J., Mali, P. & Church, G. M. *CasFinder: Flexible algorithm for identifying specific Cas9 targets in genomes*. (2014). doi:10.1101/005074

10. Langmead, B., Trapnell, C., Pop, M. & Salzberg, S. L. Ultrafast and memory-efficient alignment of short DNA sequences to the human genome. *Genome Biol.* **10,** R25 (2009).

11. Mali, P. *et al.* RNA-guided human genome engineering via Cas9. *Science* **339,** 823–6 (2013).

12. Karolchik, D. *et al.* The UCSC Genome Browser database: 2014 update. *Nucleic Acids Res.* **42,** D764–70 (2014).

13. Drmanac, R. *et al.* Human genome sequencing using unchained base reads on self-assembling DNA nanoarrays. *Science* **327,** 78–81 (2010).

14. Ball, M. P. *et al.* A public resource facilitating clinical use of genomes. *Proc. Natl. Acad. Sci. U. S. A.* **109,** 11920–7 (2012).

15. Li, J. B. *et al.* Multiplex padlock targeted sequencing reveals human hypermutable CpG variations. *Genome Res.* **19,** 1606–15 (2009).

16. Jiang, H. & Wong, W. H. SeqMap: mapping massive amount of oligonucleotides to the genome. *Bioinformatics* **24,** 2395–6 (2008).
